# Comparison of the Efficacy of Adding Clonidine, Chlorpromazine, Promethazine, and Midazolam to Morphine Pumps in Postoperative Pain Control of Addicted Patients

**DOI:** 10.5812/kowsar.22287523.1336

**Published:** 2011-07-01

**Authors:** Farnad Imani, Poupak Rahimzadeh, Seyyed Hamid Reza Faiz

**Affiliations:** 1 Department of Anesthesiology and Pain Medicine, Rasoul-Akram Medical Center, Tehran University of Medical Sciences, Tehran, IR Iran; 2 Department of Anesthesiology, Rasoul-Akram Medical Center, Tehran University of Medical Sciences, Tehran, IR Iran

**Keywords:** Patient-controlled Analgesia, Addictive Behavior, Clonidine, Morphine, Chlorpromazine

## Abstract

**Background:**

Addicted patients present difficulties for pain management because they have another problem besides their pain. Adding adjuvants to opioid pumps to intensify quality, control other problems, lengthen analgesia, and reduce side effects has been considered in the field.

**Objectives:**

The objective of this study was to evaluate the analgesic effects of adding clonidine, promethazine, chlorpromazine, and midazolam to morphine in patient-controlled intravenous analgesia (PCIA) in orthopedic patients with addiction problems.

**Patients and Methods:**

90 patients with histories of substance abuse were enrolled in this randomized controlled trial. Patients were randomly divided into three groups. The first group received 20 mg of morphine sulfate +50 mg of chlorpromazine + 50 mg of promethazine +10 mg of midazolam (M20P). The second group received the first group’s regimen plus 150 micrograms of clonidine (M20PC). The third group received 40 mg of morphine sulfate (M40). A pump with a flow rate of 5 mL/h was chosen. Patients were evaluated every 12 hours, and VAS, VRS, extra opioid usage, nausea and vomiting, and sedation scores were recorded.

**Results:**

Patients’ nausea and vomiting and sedation scores were not statistically different between the three groups. Mean VAS and VRS scores were found to be statistically lower in the M20PC group than in the other groups. Extra opioid usage between the three groups was statistically lower in the M20PC group than in the other groups. The percentage of patients satisfaction was significantly higher in the M20PC group than in the other two groups.

**Conclusions:**

This study showed that, compared to simply increasing the dose of morphine, adding chloropromazine, promethazine, midazolam, and clinidine to morphine significantly controlled pain scores and increased treatment satisfaction in addicted patients without notable side effects.

## 1. Background

Pain control in addicted patients has always been very challenging for pain physicians, especially in cases of acute pain in the postoperative period. Because of the unpredictability in achieving a satisfactory response to any given dose of opioid (1). All pain, whether acute or chronic, has three experiential components: the physical or nociceptive component, the affective or mood component, and the functional component. It is sometimes difficult for patients with addictive disorders and for their physicians to distinguish which aspect of the patient’s distress represents pain and which represents opioid craving (2). Acute pain is often associated with autonomic responses such as increases in blood pressure and heart rate, sweating, or skin blanching. Typically, it is accompanied by a mood state of anxiety or diminished function. Addicted individuals may have intermittently high levels of sympathetic arousal, either as a result of withdrawal or due to direct sympathetic stimulation by the drugs abused. Such sympathetic stimulation may alter nociceptive pathways and pain-inhibiting mechanisms in ways that intensify the pain experience. On the other hand, patients often fear increased pain when their usage is discontinued. Undertreatment of acute pain (3, 4) may be more common in individuals with addictive disorders (5). The reasons for this appear to be related to fears of causing or exacerbating addiction through the use of opioids. If a patient has an opioid addiction, the clinician should not consider opioid discontinuation until the acute pain situation is resolved (6). Therefore, in acute pain service for addicted patients, opioids are the mainstay of treatment, and they should be used in effective doses. Addicted patients often are experts on the drug doses they require to meet their basic dependence needs and the additional levels required to treat their acute pain.

Patient controlled analgesia (PCA) is a useful method for providing pain relief in opioid-addicted patients because their opioid requirement is often higher than average and because it helps to avoid staff–patient confrontation over analgesia. Also, the PCA bolus dose may have to be increased to achieve the desired analgesic effect.

## 2. Objectives

Given the difficulties associated with pain medication for addicted patients, we used and compared different drug regimens in patients with PCA.

## 3. Patients and Methods

In a randomized prospective, single-blind, clinical trial study, 90 patients with histories of substance abuse were selected for orthopedic surgery, signed a letter of consent, and were accepted into the study. The pain control route was PCA via using auto fusers at a flow rate of 5 mL/h. Inclusion criteria for study entrance were history of substance abuse > 1 year, age between 20 and 50 years, and orthopedic surgery on lower limbs. Exclusion criteria were any kind of limitation in using studied drugs; having major cardiac, renal, lung, or liver diseases; using another type of analgesia method, and patient refusal. After the operation and full recovery, a PCA pump was connected for pain control. Using a random number table, patients were randomly divided into three groups. The first group received 20 mg of morphine sulfate plus 50 mg of chlorpromazine plus 50 mg of promethazine plus 10 mg of midazolam (M20P). The second group received the first group’s regimen plus 150 micrograms of clonidine (M20PC). The third group received 40 mg of morphine sulfate (M40). The rest of the pumps were filled with normal saline up to 100 mL with a flow rate of 5 mL/h. VAS, VRS, sedation, and N&V scores; patient satisfaction; and extra opioid usage were measured (Table 1) and recorded in prepared questionnaires at 12, 24, 36, and 48 hours. Patients with a VAS score above 3 received intravenous injections of 4 mg of morphine. In the cases with an N&V score over 3 and respiratory depression and sedation scores over 2, the pump was stopped for 12 hours. The statistical analysis was carried out with one-way ANOVAs in SPSS 11.5. Duncan’s and post-hoc tests were then used to separate means.

**Table 1.** VAS, VRS, sedation, and N&V scores; patient satisfaction; and extra opioid usage (Pain score from zero to ten)

**VAS ^a^**	0 = No pain
	10 = The worst imaginable pain
**VRS ** ^**b**^	1 = No pain
	2 = Mild pain
	3 = Moderate pain
	4 = Severe pain
**Sedation score**	0 = Restless
	1 = Calm
	2 = Sleepy
	3 = Drowsy with response to verbal stimuli
	4 = Drowsy without response to verbal stimuli
	5 = Without response to painfull stimuli
**N&V score ** ^**c**^	1 = No N&V
	2 = Mild nausea without drug need
	3 = Nausea with drug need
	4 = No response to one drug attempt
**Satisfaction score**	1 = Excellent
	2 = Good
	3 = Moderate
	4 = Poor

^a^ VAS = visual analogue scale

^b^ VRS = verbal rating scale

^c^ N&V = nausea and vomiting score

## 4. Results

Ninety male patients were included in the study. Demographic findings of the study (age, weight, height, operation duration, opioid type, and opioid usage type) are shown in Table 2. No significant statistical difference was observed between the three groups. The main findings of this study such as VAS, sedation score, mean opioid consumption, and patient satisfaction are presented in Table 3. The N&V and sedation scores were not statistically different between the three groups (p = 0.1 and 0.12, respectively). Mean VAS and VRS in the first day were evaluated in the three groups by Duncan’s and one-way ANOVA tests and were found to be statistically lower in the M20PC group than in the other groups (p = 0.01 and 0.05, respectively). Mean VAS and VRS on the second day in the three groups were evaluated by Duncan’s and one-way ANOVA tests and were statistically lower in the M20PC group than in the other groups (p = 0.01 and 0.05, respectively). Extra-opioid usage in the first and second day between the three groups were evaluated using Duncan’s tests, and usage was statistically lower in the M20PC group than in the other groups (p = 0.01 and 0.05, respectively). Total opioid consumption was measured and evaluated by post-hoc tests, which revealed statistically significant differences between the first two groups and the M40 group; specifically, total opioid consumption was lower in the M20P and M20PC groups (p = 0.01), but the percentage of patients who were satisfied with their pain medication was higher in the M20PC group than in the other two groups (p = 0.05).

**Table 2.** Demographic findings in the three study males groups (n = 30)

	Morphine (20 mg) plus Protocol (M20P)	Morphine (20 mg) plus Protocol plus Clonidine (M20PC)	Morphine (40 mg )(M40)	P value
**Age (y) (Mean ± SD)**	36 ± 10	38 ± 11	39 ± 10	0.3
**Height (cm) (Mean ± SD)**	172 ± 4	170 ± 10	167 ± 9	0.2
**Weight (kg) (Mean ± SD)**	71 ± 10	68 ± 11	72 ± 12	0.23
**Operation time (h) (Mean ± SD)**	3.4 ± 0.9	3 ± 0.5	3.7 ± 0.3	0.12
**Opioid type (%)**				
**Natural**	82	76	78	0.14
**Others**	18	24	22	0.2
**Usage type (%)**				
**Inhalation**	65	54	60	0.1
**Oral**	25	30	26	0.12
**Injection**	10	16	14	0.14

**Table 3.** All findings in the three groups (VAS, VRS, N&V score, sedation, opioid consumption, and patient satisfaction)

Variables	Morphine (20 mg) plus Protocol (M20P)	Morphine (20 mg) plus Protocol plus Clonidine (M20PC)	Morphine (40 mg) (M40)	P value
**VAS ^a^**				
**Day 1 (Mean ± SD)**	25 ± 2	21.5 ± 2	27.5± 2.8	0.01
**Day 2 (Mean ± SD)**	24 ± 2.5	20.2 ± 2.2	27 ± 1	0.01
**Total (Mean ± SD)**	24.5± 2.5	20.6± 1.5	27.2 ± 1.3	0.05
**VRS ** ^**b**^				
**Day 1 (Mean ± SD)**	2.1± 0.6	1.3 ± 0.6	2.3 ± 0.6	0.05
**Day 2 (Mean ± SD)**	2.1 ± 0.5	1.4 ± 0.5	2.3 ± 0.5	0.01
**Sedation score**				
**Day 1 (Mean ± SD)**	1.17 ± 0.2	1 ± 0.2	1.2 ± 0.2	0.12
**Day 2 (Mean ± SD)**	1.2 ± 0.13	1.04 ± 0.1	1.2 ± 0.1	0.11
**N&V ** ^**c**^				
**Day 1 (times) (Mean ± SD)**	1.47 ± 0.2	1.27 ± 0.6	1.37 ± 0.2	0.13
**Day 2 (times) (Mean ± SD)**	1.37 ± 0.2	1.46 ± 0.3	1.29 ± 0.4	0.12
**Extra opioid**				
**Day 1 (mg) (Mean ± SD)**	3.5 ± 0.4	2.2 ± 0.3	4.1 ± 0.2	0.05
**Day 2 (mg) (Mean ± SD)**	3.3 ± 0.2	2.1 ± 0.3	4 ± 0.1	0.05
**Total opioid usage (mg) (Mean ± SD)**	47 ± 0.4	43 ± 0.3	86.5 ± 0.9	0.01
**Patient satisfaction (%)**	70	85	55	0.05

^a^ VAS: visual analogue scale

^b^ VRS: verbal rating scale

^c^ N&V: nausea and vomiting score

## 5. Discussion

Chronic use of opioids, alcohol, cocaine, and other drugs has been reported to induce various changes in central opiate receptors and in norepinephrine, serotonin, dopamine, and GABA availability, altering neuromodulation of brain-reward mechanisms. Such receptor and neurotransmitter changes may affect modulation of nociception as well (7). Effective pain-treatment interventions in addicted patients vary according to the physiologic causes of pain, other symptoms, and distresses associated with pain and their psychological problems. Patients in certain situations, such as those who take other nonopioid drugs unrelated to their addiction, may have special needs that should be considered when designing their treatment therapy (8). Creating effective pain management is not acheiveble by increasing opioid dosage alone because such an approach is not only unable to improve patient satisfaction or comfort or to control their symptoms, but it also might make them prone to further side effects and of course opioid tolerance. For these reasons, previous research has strongly recommended to use adjuvant therapy in pain control of patients with substance abuse (9). Therefore, in treating pain in addicted patients, multiple medications may be used to reduce pain and to manage distressing sequelae and perpetuating factors, such as sleep disturbance, restlessness, anxiety, depression, and craving (10-12). Some medical professionals who specialize in medication for addicted patients have described a “syndrome of pain facilitation or disinhibition” as occurring in the presence of a painful and actively addictive disease. This situation is characterized by a diffuse anatomic pattern of a relatively constant level of pain and a lack of response to any intervention other than the administration of the chemical on which the individual is dependent (13, 14). Changes in opiate receptors and in endogenous systems of pain inhibition definitely play important roles in this observed phenomenon in some individuals who are chronically dependent (7). The results of the present study showed that, instead of simply increasing the dosage of morphine, using morphine in addition to chlorpromazine, promethazine, midazolam, and clinidine significantly controlled pain scores and increased patient satisfaction without having notable side effects. The VRS and VAS scores of the patients in the M20PC group were significantly lower than in the other two groups, and total opioid consumption dosage was much lower, especially in comparison with the M40 group. Still, patient satisfaction with the drug regimen was higher in the M20PC group. Morphine is a strong mu-agonist with long-acting effects, playing a major role in treating the pain of addicted patients. Chlorpromazine, a phenothiazine with antipsychotic and anti-vomiting effects, (15) has been used for acute migraine attacks. Chlorpromazine has also been used as a useful adjuvant for treating withdrawal symptoms in heroin-addicted patients. Also, research has shown that chlorpromazine plays a substantial role in craving decline in addicted patients by inhibiting the postsynaptic dopaminergic mesolimbic receptors (16-18). Promethazine is useful in treating nausea, vomiting, and motion sickness. It has been used for chronic pain attacks. Additionally, promethazine and some antihistaminic drugs have been found to play effective roles in treating withdrawal symptoms (19, 20). Midazolam is a short-acting benzodiazepine and has central analgesic effects by inhibiting glutamate receptors in cord. Also, animal studies have revealed the effects of benzodiazepines in the prevention of opioid tolerance (especially morphine) and drug dependency (21). Although the analgesic role of alpha-2 adrenoceptor agonists in opioid dependency has been described in the literature (8), few clinicians appear to make regular use of this approach. The membrane-based opioid receptor and the alpha-2 receptor share similarities in both being part of a large superfamily of G-protein-coupled receptors. Activation of the G-protein-coupled receptor initiates interaction with an inhibitory G-protein, resulting in a reduction in neurotransmission, which is expressed in the individual as the quietening that is typically seen after morphine or clonidine use (9). Clonidine provides analgesia after surgery or trauma and is particularly useful when opioid withdrawal may be complicating the situation, acting synergistically with any background opioid but conveniently not promoting nausea, vomiting, or respiratory depression. Also, clonidine could decrease craving in addicted patients (22). Heavy users of opioids may demonstrate a degree of resistance to parenteral clonidine, but a 2-4 µg/kg intravenous dose of the drug will usually produce a noticeable quietening and sedation of the patient in 5 to 10 minutes (8). On the other hand, patient-controlled-analgesia pumps have many advantages that make their use ideal for addicted patients. In particular, such pumps can prepare adequate serum concentration levels in the therapeutic window range, result in less fluctuation in the serum level of the pain medication, create better overall analgesic effects, and lower the total amount of drug consumption with better patient satisfaction. Ultimately, to control addicted patients’ pain, the consensus in the field is to maintain regular provision of the patient’s preexisting opioid requirement, with additional analgesia. Ideally, a multimodal approach, with appropriate combinations of local anesthesia, alpha-2 agonists, anti-histaminic, benzodiazepines, antidopaminergics, ketamine, anti-inflammatory analgesics, and paracetamol is advocated (23, 24). Using higher bolus doses with PCA and shorter lock-out intervals is a recommended strategy (25). Looking at this issue from different perspectives, it is clear that this regimen could be a suitable choice if further studies confirm the present study’s finding or provide similar results for other drugs in the same family. In summary, considering all of the above mentioned findings, it seems reasonable and quite worthy to add chlorpromazine, promethazine, midazolam, and clonidine to morphine for suitable control of pain and other problems of addicted patients in acute pain management. The authors of this article advocate more research and trials on this issue to identify the most effective drug regimen for patients with substance-abuse problems.
